# Can Google Glass facilitate work for nurses in the emergency department?

**DOI:** 10.1186/1757-7241-23-S1-A53

**Published:** 2015-07-16

**Authors:** Thomas Schmidt, Charlotte Mose Hansen, Jan Dahlin

**Affiliations:** 1 The Mærsk Mc-Kinney Møller Institute, University of Southern Denmark, Odense M, Denmark; 2 Emergency Department, OUH Odense University Hospital, Odense C, Denmark

## Background

One of many challenges for Emergency Departments is having access to clinical guidance when and where it is needed. In this abstract we provide a first experience from a pilot study involving the use of Google Glass in the Emergency Department among nurses. The involved nurses were asked to evaluate the experience of using Google Glass as a communication device instead of traditional mobile phones.

## Methods

Each participant responded to a two-part survey with two descriptive and four Likert scaled questions. Responses were analyzed in Excel through descriptive analysis of the survey responses. The descriptive section asked for clinical role, number of usage attempts, and number of patients attended to. The Likert questions where: 1) Experience of call quality, 2) Experience of sound quality, 3) Total usage experience, and 4) Patient interaction experience. Each of these questions where scaled in five steps from poor, below average, average, above average, good, and grouped into below, average, and above in the analysis.

## Results

11 of 12 involved nurses responded to the survey, with a role distribution of 1 triage shift, 4 receiving ward shifts, 2 coordinating nurse shifts, 3 shifts treating fast track injuries, and one who covered several of these roles. The average nurse attended to 9.9 patients, and on average attempted to use the equipment 5.5 times during each shift. 55% experienced call quality as below average, 36% as average, and 9% as above average. 73% experienced sound quality as below average, 18% as average, and 9% above average. 64% marked total usage experience as below average, 27% as average, and 9% above average. 64% said patient interaction experience was below average, 27% as average, and 9% above average.

## Conclusion

The main obstacle of using Google Glass was issues with the quality of sound. Usability measures such as total usage and patient interaction experience may have scored higher had experience with sound quality been better. Thus, given the ubiquitous and individual nature of wearable technology, further studies should be made.

